# ATP13A2 (PARK9) and basal ganglia function

**DOI:** 10.3389/fneur.2023.1252400

**Published:** 2024-01-05

**Authors:** Kristina M. Croucher, Sheila M. Fleming

**Affiliations:** ^1^Department of Pharmaceutical Sciences, Northeast Ohio Medical University, Rootstown, OH, United States; ^2^Biomedical Sciences Graduate Program, Kent State University, Kent, OH, United States

**Keywords:** Parkinson’s disease, Kufor-Rakeb Syndrome, neuronal ceroid lipofuscinosis, manganese, iron, zinc, mitochondria, alpha-synuclein

## Abstract

ATP13A2 is a lysosomal protein involved in polyamine transport with loss of function mutations associated with multiple neurodegenerative conditions. These include early onset Parkinson’s disease, Kufor-Rakeb Syndrome, neuronal ceroid lipofuscinosis, hereditary spastic paraplegia, and amyotrophic lateral sclerosis. While *ATP13A2* mutations may result in clinical heterogeneity, the basal ganglia appear to be impacted in the majority of cases. The basal ganglia is particularly vulnerable to environmental exposures such as heavy metals, pesticides, and industrial agents which are also established risk factors for many neurodegenerative conditions. Not surprisingly then, impaired function of ATP13A2 has been linked to heavy metal toxicity including manganese, iron, and zinc. This review discusses the role of ATP13A2 in basal ganglia function and dysfunction, potential common pathological mechanisms in ATP13A2-related disorders, and how gene x environment interactions may contribute to basal ganglia dysfunction.

## Introduction

**Figure 1:**
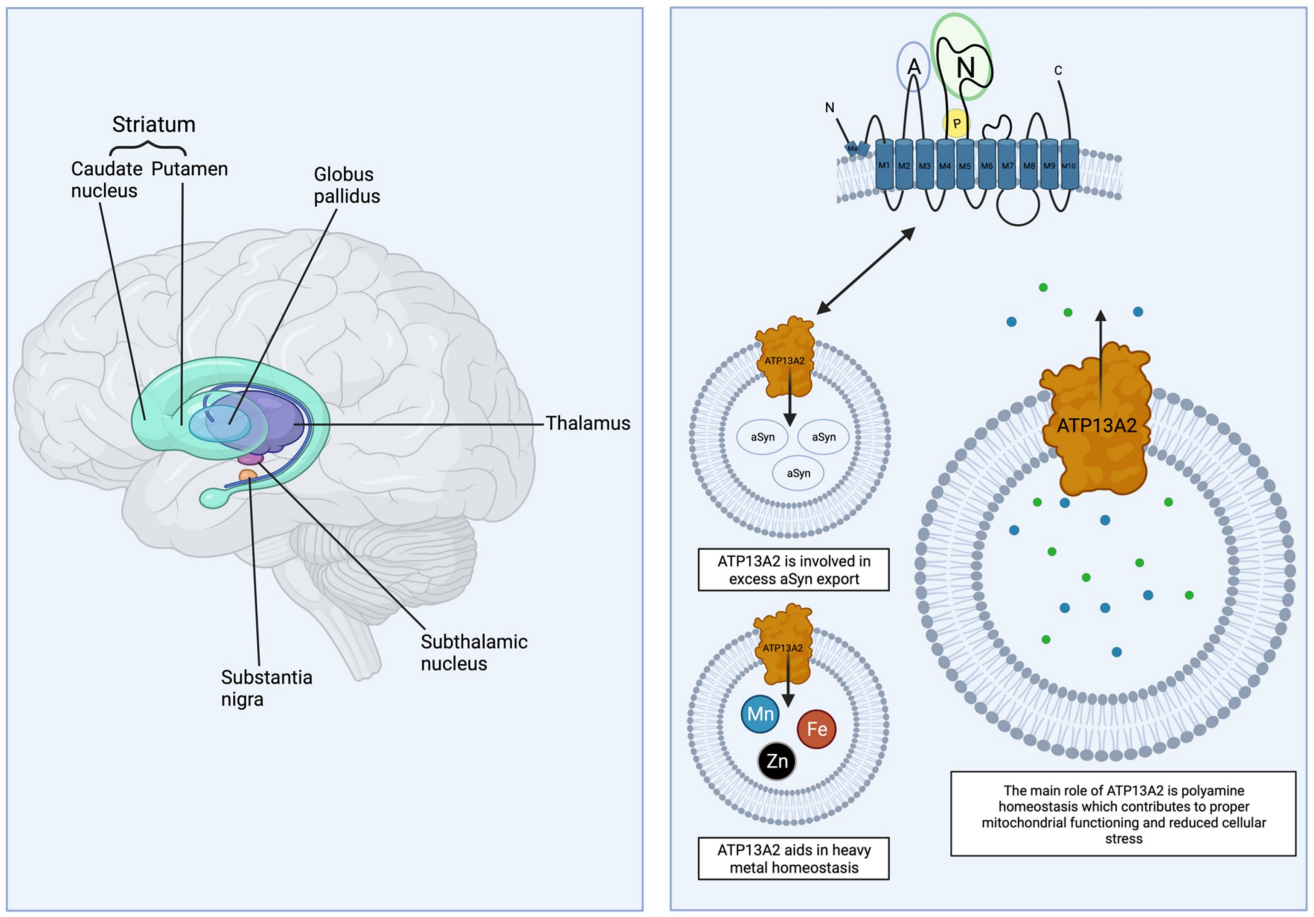
ATP13A2 and the basal ganglia. The basal ganglia and related nuclei (striatum, globus pallidus, subthalamic nucleus, thalamus, and substantia nigra) are vulnerable to genetic and environmental factors. Wildtype ATP13A2 is a protein with 10 transmembrane domains localized to the lysosomal membrane and is involved in polyamine transport and homeostasis, alpha-synuclein export, and intracellular heavy metal regulation. Small green and blue dots represent the polyamines spermidine and spermine. Created with BioRender.com.

ATP13A2 is an ATPase primarily located in early and late endosomes and lysosomes. Biallelic mutations in the gene *ATP13A2* cause Kufor-Rakeb Syndrome (KRS; OMIM#606693), also known as Parkinson’s disease-9 (PARK9), a juvenile form of Parkinson’s disease (PD) (1). KRS patients typically develop Parkinsonian motor symptoms and show some degree of levodopa-responsiveness (1). Following KRS, *ATP13A2* was determined to be mutated in forms of neuronal ceroid lipofuscinosis (NCL), hereditary spastic paraplegia (HSP), and most recently amyotrophic lateral sclerosis (ALS) (2–7). Genetic analysis also shows that *ATP13A2* variants in *LRRK2* (PARK8) G2019S carriers, the most common cause of hereditary PD, are common and may modify disease onset and severity (8). In idiopathic PD and Dementia with Lewy bodies, *post mortem* analysis shows ATP13A2 protein levels are significantly decreased suggesting altered ATP13A2 function may be more pervasive in phenotypic PD than previously thought (9). Loss of function of *ATP13A2* has also been linked to an increased sensitivity to heavy metal toxicity including manganese, iron, and zinc (10–23). Given the diverse outcomes that can result from dysfunctional ATP13A2, it is important to determine commonalties between these disorders in terms of symptom expression, peripheral and central pathology, and mechanisms of neurodegeneration in order to identify novel therapeutic strategies and targets. Currently, there is limited human pathology data on ATP13A2-related disorders but analysis of the different clinical profiles point to the basal ganglia as the central network disrupted in the majority of cases (24–32). The basal ganglia and its network (Figure 1) are particularly vulnerable to neurodegeneration and are associated with genetic and environmental factors that drive disorders such as PD, dystonia, and Huntington’s disease, among others (33). In addition, the basal ganglia are important in heavy metal transport with multiple structures negatively impacted by excessive intake, including manganese and iron. Thus, understanding how ATP13A2 contributes to basal ganglia function will be essential for the identification and development of therapeutics for ATP13A2-related disorders.

## Clinical syndromes and ATP13A2

### Kufor-Rakeb Syndrome

Mutations in *ATP13A2* are linked to the juvenile parkinsonism KRS. KRS is an autosomal recessive form of PD with similar but distinct neurological symptoms and neurodegeneration (1, 34). It was first identified in five members of a consanguineous family from Kufor-Rakeb, Jordan, with the youngest age of onset at 11 years old (30). Symptoms of KRS originally included rigidity, bradykinesia, supranuclear gaze palsy, and dementia (30). In general, KRS symptom onset occurs in young patients and the condition progresses rapidly (35). MRI brain imaging shows generalized brain atrophy beginning in the globus pallidus and pyramidal tract (30). Many KRS patients respond to levodopa, suggesting nigrostriatal dysfunction similar to what is observed in sporadic PD (30, 35). Patient follow-up performed 10 years later showed similar symptoms, but now with the addition of myoclonus and increased pyramidal signs. At the time of these studies, the link between KRS and *ATP13A2* had not been made (32). Later, KRS was also identified in a Chilean population and symptoms were described in a longitudinal study (1, 24). Five family members were diagnosed with KRS between the ages of 10 and 13 with early symptoms of rigidity, frequent falls, slowed movement and speech, abnormal gait, cognitive impairment, insomnia, and upward gaze palsy. The progression of these symptoms was slower than that seen in the Jordanian family (1). Years after diagnoses, bradykinesia, resting tremor, spasticity, and myoclonus, developed. Brain imaging revealed generalized atrophy and hypointensity within the basal ganglia (24).

The genomes of the Jordanian and Chilean families were later screened to identify the genetic locus of the mutations (1). In the Jordanian family, patients had a homozygous duplication of 22 base pairs in exon 16 resulting in a frameshift and a premature stop codon (c.1632_1653dup22/p.Leu552fsTer788). In the Chilean family two compound heterozygous mutations were identified, a deletion of cytosine at the nucleotide position 3,057 in exon 26 causing a frameshift mutation (c.3057delC/p.1019GfsX1021) and a transition from guanine to adenine at the +5 position of the donor splice site in exon 13 (c.1306 + 5G > A/p.G399_L435del) (1). These mutations resulted in a loss of function of *ATP13A2* which was then classified as a familial form of PD, PARK9. Since these studies, additional *ATP13A2* mutations in various populations have been identified including a homozygous c.1510G > C/p.Gly504Arg mutation and the heterozygous mutations c.35C > T/p.Thr12Met or c.1597G > A/p.Gly533Arg (Table 1) (27, 39, 41). Similar to the earlier cases, patients developed basal ganglia related symptoms such as bradykinesia, rigidity, and levodopa responsiveness (27, 39, 41). Diffuse atrophy of the brain, supranuclear gaze palsy, and postural instability were observed in homozygous mutations (27). While in heterozygous mutations (ex. c.2236G > A/p.Ala746Thr), symptoms varied, but included basal ganglia-related bradykinesia, rigidity, and tremor (36–38, 40). In general, the homozygous mutations appear more severe compared to the heterozygous mutations, but symptoms can still appear in the heterozygous state with later age of onset. Further research regarding the heterozygous c.35C > T/p.Thr12Met, c.1597G > A/p.Gly533Arg, and c.2236G > A/p.Ala746Thr mutations is needed to better understand their pathogenicity, as KRS is an autosomal recessive disorder (Table 1) (1, 24, 27, 30, 35–37, 40).

**Table 1:** Clinical syndromes associated with mutations in *ATP13A2*.

Syndrome	Mutations (RefSeq: NM_001141973.3)	Age (years)	Clinical symptoms	Imaging pathology	Postmortem pathology	References
Kufor-Rakeb	c.35C > T/p.Thr12Met, c.546C > A/p.Phe182Leu, c.701G > A/p.Arg294Gln, c.746C > T/p.Ala249Val, c.844A > T/p.Ser282Cys, c.1306 + 5G > A/p.G399_L435del, c.1346G > A/p.Arg449Gln, c.1510G > C/p.Gly504Arg, c.1597G > A/p.Gly533Arg, c.2236G > A/p.Ala746Thr, c.2473C > AA/p.Leu825fs, c.2629G > A/p.Gly877Arg, c.2762C > T/p.Gln858*, c.2836A > T/p.Ile946Phe, c.2939G > A/p.Arg980His, c.3176 T > G/p.Leu1059Arg, c.3274A > G/p.Gly1014Ser, c.1346G > A/p.Arg449Gln, c.1108_1120del13/p.Arg370fsX390, c.2742_2743delTT/p.F851CfsX856, c.3057delC/p.1019GfsX102, c.3253delC/p.L1085wfsX1088, c.1103_1104insGA/p.Thr367fsX29	10–29 (hom) 5, 20–70 (het)	Rigidity, bradykinesia, resting tremor, abnormal gait, levodopa responsive, myoclonus, supranuclear gaze palsy	Brain atrophy, starting with the globus pallidus and pyramidal tract.	-Lipofuscin accumulation in BG, CTX, HPC, AMG, CBL, BS -Iron deposits in BG, loss of DA neurons in SNc	(24, 27–29, 31, 32, 34, 36–44)
Neuronal Ceroid Lipofuscinosis	c.2429C > G/p.Met810Arg	13–16	Rigidity, akinesia, resting tremor, dysarthria, dysphagia, impaired coordination, levodopa responsive, and cognitive impairment	–	-Neuronal and glial lipofuscinosis in CTX, basal nuclei, CBL, and retina	(2)
Hereditary Spastic Paraplegia	c.364C > T/p.Gln122Ter, c.1330C > T;3404C > T/p.Arg444Ter; Gln1135Ter, c.1535C > T/p.Thr512Ile, c.2126G > C/p.Arg709Thr, c.2158G > T/p.Gly720Trp, c.2629G > A/p.Gly877Arg, c.2675G > A/p.Gly892Asp, c.3017_3019del/p.Leu1006-Leu1007del	11–36	Spasticity and weakness, bradykinesia, cognitive impairment, slow vertical eye movements, seizures	Overall atrophy	-Corpus callosum thinning -Overall atrophy	(3–5)
Amyotrophic Lateral Sclerosis	c.1233C > G/p.Ile411Met, c.1837G > A/p.Glu613Ter	32	Limb weakness and rigidity, spastic-ataxic gait, dysphonia, cognitive impairment	-Atrophy in CBL -Motor axon neuropathy -Reduced DAT in Str	–	(7)

AMG, amygdala; BG, basal ganglia; BS, brain stem; CBL, cerebellum; CTX, cortex; HPC, hippocampus; SNc, substantia nigra pars compacta.

Recently, the first and only postmortem KRS study was completed on a patient with a homozygous *ATP13A2* missense mutation (34). In this case, symptoms appeared at approximately 12 years of age and included rigidity and akinesia, upward gaze palsy, and spasticity. Later in life, the patient suffered from severe levodopa-induced dyskinesias, hallucinations, and irritability. Postmortem analysis revealed loss of pigmented neurons in the substantia nigra, lipofuscin accumulation in many brain regions including basal ganglia, iron accumulation in basal ganglia, and temporal lobe atrophy. This offers the first confirmation of basal ganglia pathology and substantia nigra degeneration in KRS (Table 1) (34).

### Neuronal ceroid lipofuscinosis

Mutations in *ATP13A2* are also linked to NCL, a lysosomal storage disorder. NCLs are a group of degenerative diseases characterized by accumulation of autofluorescent lysosomal storage material within lysosomes (2, 6, 45, 46). NCL symptoms can include basal ganglia dysfunction, seizures, visual impairments, cerebellar ataxia, and dementia (2, 6, 45, 46). A homozygous mutation in *ATP13A2* (c.2429C > G/p.Met810Arg) was identified in a Belgian family with NCL (2). Symptoms included akinesia and rigidity in addition to gait impairments, myoclonus, and alterations in mood. Similar to KRS, levodopa responsiveness was noted along with the development of levodopa-induced dyskinesias (2). Postmortem analysis revealed widespread lipofuscinosis throughout the brain in neurons and glia (Table 1) (2).

### Hereditary spastic paraplegia

HSP is a neurodegenerative condition characterized by progressive limb spasticity (3–5). Similar to both KRS and NCL, the clinical presentation can be quite heterogenous where, in addition to limb spasticity, seizures and cognitive impairment can also develop (3–5). The first family identified with ATP13A2-associated HSP showed a variety of symptoms in addition to adult-onset of limb spasticity, with some developing bradykinesia and rigidity, cognitive deficits, and supranuclear gaze palsy (4). Brain imaging revealed cerebellar and cortical atrophy and in one case decreased dopamine transporter density in the putamen (4). Since then, several families have been identified with ATP13A2-related HSP (3–5). Again, symptoms vary but can include bradykinesia, resting tremor, neuropsychiatric dysfunction, cognitive impairments, dysarthria, dysphagia, and oculomotor impairments in addition to limb spasticity and cerebellar symptoms (Table 1) (3–5).

### Amyotrophic lateral sclerosis

Most recently, mutations in *ATP13A2* have been linked to ALS (7, 47). ALS is characterized by progressive degeneration of motor neurons leading to motor weakness, impaired breathing, and ultimately death (48). Two mutations in *ATP13A2*, c.1837G > A/p.Glu613Ter and c.1233C > G/p.Ile411Met, were identified in two family members, resulting in *ATP13A2* loss of function (7). These cases presented with limb spasticity, dysphonia, ataxic gait, and cognitive impairment. Initially, they were diagnosed with HSP but as the condition progressed, ALS-related symptoms developed. While brain imaging showed cerebellar atrophy, dopamine transporter analysis revealed a bilateral reduction in uptake in the putamen (Table 1) (7). Thus, despite the heterogenous nature of clinical symptoms and pathology in ATP13A2-associated diseases, the basal ganglia are affected in the majority of the cases.

## ATP13A2 expression and function

### Expression

P-type ATPases are a large family of proteins involved in the transport of cations and other substrates across cell membranes through the utilization of energy from ATP hydrolysis (49). Of these, P5-type ATPases are only expressed in eukaryotes and are the least characterized of the P-type ATPases. Of the P5-types, ATP13A2 is most abundant in the brain (49). Although there are limited studies on *ATP13A2* expression in the human brain, high expression in neurons in the ventral midbrain including the substantia nigra and in the basal ganglia (globus pallidus and putamen), cortex, and hippocampus has been shown (1). However, more work is needed to identify expression levels in different brain regions across species. *In vitro* studies show that ATP13A2 localizes to intracellular vesicular compartments including lysosomes and early and late endosomes implicating it in protein handling and degradation (1, 12, 21, 50, 51).

### Lipid switch

ATP13A2 is a 1,180 amino acid ATPase with 10 transmembrane domains (1, 35). Molecular analysis shows ATP13A2 is a P5B-type ATPase with the N- and C- termini residing in the cytosol (Figure 1). The ATP13A2 N-terminus hydrophobic Ma region does not span the membrane and remains cytosolic (52, 53). The N- terminus and the Ma domain are important for targeting of ATP13A2 to lysosomes as they are hydrophobic. This hydrophobicity encourages interactions with lipids, specifically phosphatidic acid (PA) and phosphatidylinositol(3,5)bisphosphate [Pi(3,5)P2], which are present at high concentrations in endosomal and lysosomal membranes (52). These two lipids bind to three distinct regions in the N-terminus, which partially includes the Ma domain, to regulate ATP13A2 activity by stimulating autophosphorylation. Although PA and PI(3,5)P2 are necessary for ATP13A2 activation, they are not the transported substrates (52). Biochemical studies show that ATP13A2 activity depends on these signaling lipids and it is important to note that both are involved in vesicular trafficking, membrane fission and fusion, and autophagy, mechanisms known to be involved in multiple neurodegenerative disorders (54–58). The conformational states of ATP13A2 have also been recently identified and will facilitate the development of targeted mechanistic therapeutics (59–62).

### Polyamine transport

The polyamines spermidine and spermine are highly regulated in cells and bind to nucleic acids to aid in optimal cell function including gene transcription and translation, cell cycle progression, oxidative stress response, and metabolism (61, 63, 64). Within the human brain, polyamine concentration decreases with age in multiple regions including basal ganglia structures (putamen, globus pallidus, and subthalamic nucleus) and cerebellum (65). Alterations in the polyamine pathway are also linked to PD (66). Studies by Pinto et al. (67) and De La Hera et al. (68) were the first to suggest ATP13A2 may be involved in polyamine transport. It is now confirmed that ATP13A2 transports the polyamines spermidine and spermine and functions as a H^+^/K^+^-ATPase to regulate polyamine levels (Figure 1) (64, 69–71). Specifically, ATP13A2 transports polyamines from the lysosome to the cytosol to maintain polyamine homeostasis (69, 71). Loss of ATP13A2 function subsequently leads to toxic polyamine accumulation within the lysosome (64). Polyamine accumulation may then impact other key cellular functions including protein degradation and mitochondrial function.

Although there are a limited number of studies on the expression profile of ATP13A2 across species, it is found to be abundant within basal ganglia structures and in regions that provide important innervation to the basal ganglia including substantia nigra and cortex.

## ATP13A2 and heavy metal susceptibility

Several heavy metals preferentially accumulate within basal ganglia structures and are linked to multiple neurodegenerative conditions (72). Heavy metal transporters such as divalent metal transporter 1 (DMT1) are abundant in basal ganglia structures and facilitate metal homeostasis (73). Excessive exposure to heavy metals and/or genetic mutations to metal transporters can impair heavy metal handling and transport leading to motor and cognitive impairments in humans (74–77). ATP13A2 function appears to be important in maintaining heavy metal balance (Figure 1) as loss of function mutations are linked to increased susceptibility to manganese, iron, and zinc toxicity.

### Manganese

Manganese (Mn) is an essential metal involved in multiple cellular functions including but not limited to energy metabolism, antioxidant response, the immune response, and development (78–80). Mn is ubiquitous in the environment and thus, Mn deficiency is rare. In contrast, excessive exposure to Mn, especially in certain occupations such as mining and welding, is a significant health risk and can cause manganism, an age-related neurodegenerative condition. Manganism is characterized by PD-like motor symptoms and cognitive impairment but is distinct from classical PD as the motor deficits are typically not responsive to levodopa and additional impairments such as dystonia and “cock-walk” gait develop. It has been shown that Mn preferentially accumulates in the basal ganglia affecting primarily the globus pallidus (74).

Mn is transported by a variety of metal transporters, including but not limited to DMT1, dopamine transporter (DAT), L-type calcium channels, transferrin, and transferrin receptor (81–83). Mn enters the brain primarily through DMT1 and transferrin/transferrin receptors [transferrin-dependent pathway; (81, 84)]. DMT1 expression in nonhuman primate brain shows high levels in the caudate nucleus, putamen, internal and external globus pallidus, and moderate expression in the substantia nigra pars compacta, thalamus and subthalamic nucleus (85). DAT is shown to transport Mn during excess exposure and is highly expressed in the striatum (86). The compounded effect of DMT1 and DAT transport of Mn during excess exposure contributes to the preferential accumulation within basal ganglia structures.

Intracellular Mn toxicity is associated with multiple mechanisms also involved in neurodegenerative diseases such as mitochondrial dysfunction, ER stress, impaired protein degradation, oxidative stress, and apoptosis (75, 87, 88). Since manganism does not develop in everyone exposed to high Mn levels, it suggests that genetic susceptibility may be an important contributing factor. Indeed, loss of function mutations in the Mn efflux transporter Slc30a10 cause an inherited form of Mn-induced Parkinsonism without excessive exposure (77). *ATP13A2* may be another genetic susceptibility factor in Mn toxicity. Polymorphisms in *ATP13A2* were shown to influence Mn toxicity in an elderly population (76). Mn toxicity and ATP132 have been extensively examined in different cell systems, yeast, and *in vivo* (Table 2). In cultured human neuroblastoma cells (NLF cell line), overexpression of *ATP13A2* results in cellular protection against high concentrations of Mn compared to mutated forms of *ATP13A2* (c.546C > A/p.Phe182Leu, c.1510G > C/p.Gly504Arg and c.1537G > A/p.Asp513Asn) (12). While in cultured rat primary cortical neurons, wildtype and c.1537G > A/p.Asp513Asn *ATP13A2* expression protect against Mn toxicity, c.1510G > C/p.Gly504Arg and c.546C > A/p.Phe182Leu mutants do not (13). In yeast, excess Mn is sequestered to the vacuole and mutated *Ypk9* (yeast homolog of *ATP13A2*) showed a higher sensitivity to Mn toxicity than cells that expressed wildtype *Ypk9* (15, 89). Similarly, *ATP13A2* overexpression in *C. elegans* dopaminergic neurons protects against Mn toxicity, further indicating an important link between ATP13A2 and Mn homeostasis in the basal ganglia and substantia nigra (23). In *Atp13a2* knockout mice, low dose Mn exposure resulted in alterations in sensorimotor function, increased accumulation of Mn in the brain, and increased insoluble alpha-synuclein in the ventral midbrain (14). Taken together, these studies suggest an important role for ATP13A2 in Mn homeostasis (Table 2).

**Table 2:** Manganese toxicity in *ATP13A2* models.

Model system	Cellular toxicity	Mitochondrial impairments	Lysosomal impairments alphaSyn pathology	References
*In vitro* cell culture (HeLa, rat primary, NLF, HEK293, N21)	-DNA fragmentation -Decreased cell viability -Apoptotic events -Protection from cellular toxicity with *ATP13A2* WT or overexpression	Mutant -Increased glutathione -Increased caspase-3 and cytochrome c WT/overexpression -Decreased glutathione -Decreased caspase-3 and cytochrome c	–	(12, 13, 21, 23)
Yeast	-Growth defects and cell death of mutant cells -Protection from cellular toxicity with *YPK9* WT or overexpression	–	–	(15, 20)
*C. elegans*	-Dopaminergic neuron degeneration, rescued with *ATP13A2* overexpression	–	–	(23)
*Atp13a2* mice treated with Mn	-Increased Mn accumulation in brain in Mn-treated *Atp13a2*	–	-Lipofuscin accumulation in SNc of Mn-treated *Atp13a2* mice -Increased insoluble alphaSyn in the ventral midbrain of Mn-treated *Atp13a2* mice	(14)

alphaSyn, alpha-synuclein; Mn, manganese; SNc, substantia nigra pars compacta.

### Iron

Iron (Fe) is an essential metal important in vital cellular functions such as oxygen transport, electron transport, and neurotransmitter synthesis (90). Iron accumulation in the brain increases with age and is found primarily in basal ganglia regions such as the globus pallidus, putamen, and substantia nigra (91, 92). Iron is transported into the brain using a similar mechanism to Mn transferrin-dependent transport. Transferrin receptors are moderately expressed in the putamen, caudate nucleus, globus pallidus, and substantia nigra in humans (85, 93). In rodents transferrin receptors are also expressed in striatum, thalamus, and cerebellum (94). Once inside the cell, Fe is then released into the cytoplasm with the help of DMT1 (81, 82, 94). In the basal ganglia, Fe is important in DNA synthesis, mitochondrial respiration, oxygen transportation, and neurotransmitter synthesis, especially dopamine.

Dysregulation of iron is associated with several neurological conditions including PD and Neurodegeneration with Brain Iron Accumulation (NBIA). NBIA involves disorders in which iron accumulates within the basal ganglia and presents with motor and cognitive symptoms including but not limited to abnormal gait, dystonia, parkinsonism, spasticity, seizures, and impaired cognitive function (95, 96). NBIA is typically diagnosed based on clinical symptoms and MRI imaging (T2*-weighted). In addition to PD, mutations in *ATP13A2* are linked to NBIA, suggesting ATP13A2-linked disorders may be considered a form of NBIA (97). For example, in a patient with a homozygous *ATP13A2* mutation (c.1103_1104insGA/p.Thr367ArgfsX29) and clinical symptoms resembling NBIA, T2*-weighted MRI analysis showed hypointensities indicative of iron accumulation in the basal ganglia (31). Iron accumulation in the basal ganglia was also reported in the Chilean family with KRS (24). Furthermore, the first postmortem analysis of KRS showed iron accumulation in the basal ganglia however, the deposits were sparse and no axonal spheroids typical of some NBIA were observed (34). Although limited, *in vitro* work indicates ATP13A2 can protect against iron toxicity supporting a potential role for ATP13A2 in iron homeostasis within the basal ganglia (Table 3) (19).

**Table 3:** Iron and zinc toxicity in *ATP13A2* models.

Heavy metal	Model system	Cellular toxicity	Mitochondrial impairments	Lysosomal impairments	alphaSyn	References
**Iron (Fe)**						
	*In vitro* cell culture (SH-SY5Y, CHO)	-Decreased cell viability -Increased Beta-hexosaminidase -Increased viability with WT *ATP13A2*	–	-Elevated cytosolic iron -Iron induced LMP -Rescued with WT *ATP13A2*	–	(18, 19)
	*C. elegans*	-Decreased lifespan in mutants, rescued with WT *ATP13A2*	-Decreased survival when exposed to rotenone	–	–	(10)
**Zinc (Zn)**						
	*In vitro* cell culture (HEK293, SH-SY5Y, hONs, rat primary, human fibroblasts, PCNs)	-Increased cell death -Reduced neurite length -LDH release -Rescued with *ATP13A2* overexpression	-Increased cytochrome c, caspase-3, ERK1, ERK2, p38 -Complex I impairments -Decreased mitochondrial membrane potential -Increased ROS production -Reduced ATP production -Increased mitochondrial fragmentation -Rescued with *ATP13A2* overexpression	-Decreased LAMP-2 and LC3II/LC3I ratio -Increased p62 -Decreased Zn-containing vesicles -Elevated lysosomal pH -Rescued with *ATP13A2* overexpression	-Increased alphaSynand p-alphaSyn -Reduced alphaSyn association with exosomes -Rescued with *ATP13A2* overexpression	(16, 17, 22, 98)
	Yeast	-Sensitivity to Zn in mutant cells -Resistant with *ATP13A2* overexpression	–	–	–	(16)
	*C. elegans*	-Reduced survival with *catp-6* deletion	–	–	–	(10, 11)

aSyn, alpha-synuclein; p-aSyn, phosphorylated alpha-synuclein; LAMP1/LAMP-2, lysosome associated membrane protein-1 and -2; LC3II/LC3I, microtubule associated protein; LDH, lactate dehydrogenase; LMP, lysosome membrane permeabilization; SNc, substantia nigra pars compacta; TH, tyrosine hydroxylase.

### Zinc

Zinc is another essential metal involved in numerous cellular processes including synthesis of DNA and proteins (17, 22). While zinc deficiency is well studied, less is understood about the mechanisms of excess and accumulated zinc (98). Zn is most notably transported by zinc-regulated zinc transporter 1, ZIP8/ZIP14, and DMT1 (81, 82). Zinc accumulation has been shown in the basal ganglia and substantia nigra in sporadic PD patients and is linked to loss of function mutations in *ATP13A2* (16, 17, 22, 95, 98–100). Analysis in PARK9 patient-associated cultures showed increased sensitivity to zinc, lysosomal dysfunction, mitochondrial alterations, and increased alpha-synuclein (Table 3). In addition, overexpression of *ATP13A2* reduced these pathological features *in vitro* (16, 22, 98, 100). While *in vitro* human-derived ATP13A2 models have been investigated, there are no imaging or postmortem studies to date to demonstrate alterations in zinc homeostasis in patients.

Taken together, clinical, *in vivo*, and *in vitro* studies suggest long-term impairment in ATP13A2 function may impair the basal ganglia’s ability to maintain metal homeostasis.

## ATP13A2 and mechanisms of neurodegeneration

Mutations in *ATP13A2* are associated with diverse disorders of overlapping symptoms and with heavy metals that share common transport mechanisms. Thus, it should expected that *ATP13A2* mutations affect key pathological systems, such as mitochondrial function and lysosome-mediated protein degradation, involved in most neurodegenerative disorders.

### Mitochondrial function

At some stage in every neurodegenerative disease there is mitochondrial dysfunction. Determining where in the brain mitochondrial dysfunction occurs, when it happens, and how it begins are critical questions for every neurodegenerative condition. Mutated *ATP13A2* is linked to multiple mitochondrial defects (Tables 3, 4). Studies in PARK9 fibroblasts and *ATP13A2* knockdown in cortical neurons collectively reveal impaired autophagic flux and the following mitochondrial defects: reduced mitochondrial membrane potential, reduced ATP synthesis, increased respiration rate, increased fragmentation, and reactive oxygen species (ROS) (107, 108). While overexpression of *ATP13A2* confers resistance against the mitochondrial complex 1 inhibitors rotenone and MPP^+^ (12). In addition, the ATP13A2 associated lipids PI(3,5)P2 and PA when pharmacologically inhibited, result in mitochondrial stress and toxicity in mutant cells exposed to rotenone (109). These data suggest loss of function mutations in *ATP13A2* are associated with mitochondrial defects that could lead to increased susceptibility to environmental insults such as heavy metal and pesticide exposures and ultimately to neurodegenerative disease.

**Table 4:** Consequences of impaired *ATP13A2* function *in vivo* in rodents.

Rodent	Manipulation	Behavior	Pathology	References
Knockout mouse	*Atp13a2* knockout	-Impairments in beam walking, gait, and spontaneous activity	-Lipofuscin accumulation in CBL, CTX, HPC -alphaSyn accumulation in HPC	(6)
	*Atp13a2* conditional knockout (brain)	-Impairments in rotarod and wire hang test	-Lipofuscin accumulation in CTX, HPC, SNc -Increased GFAP and subunit c in CTX -Reduced cathepsin D in CTX	(101)
Heterozygote mouse	*Atp13a2* heterozygous and knockout	N/A	*Atp13a2* mice -Lipofuscin accumulation in CTX, HPC, CBL, BS -Increased ubiquitin inclusions -Increased GFAP in CTX, HPC, CLB -Increased Iba-1 in CTX, HPC, CBL, BS *Atp13a2* Het -Lipofuscin accumulation in CTX -Increased GFAP and Iba-1 in CTX, HPC, BS	(102)
	*Atp13a2* Heterozygous and alphaSyn preformed fibrils (PFFs)	-Impairments in olfaction	-Increased microglia	(103)
*Atp13a2* mice with alphaSyn overexpression	*Atp13a2* combined with overexpression of A53T alphaSyn	-Impairments in rotarod and open field test	-Increased lipofuscin and gliosis in the CTX, CBL, Str, HPC, THL in *Atp13a2* mice -Increase in LAMP1, LAMP2, and BMP in *Atp13a2* -Altered cathepsin D in *Atp13a2*	(104)
	*Atp13a2* combined with overexpression of WT alphaSyn	-Enhanced sensorimotor alterations in tests of locomotor and spontaneous activity, beam walking, and adhesive removal	N/A	(105)
*Atp13a2* Mouse	*Atp13a2* mice with ischemic stroke	N/A	-Increased LC3-II in the CTX -Increased expression of Bax and caspase-3	(106)
*Atp13a2* mouse	*Atp13a2* mice treated with low dose Mn	-Enhanced beam walking, gait, and spontaneous activity in Mn-treated *Atp13a2* mice -Impairments in locomotor and spontaneous activity in *Atp13a2* mice	-Lipofuscin accumulation in PFC, CBL, HPC in *Atp13a2* mice -Lipofuscin accumulation in SNc of Mn-treated *Atp13a2* mice -Increased insoluble alphaSyn in ventral midbrain in Mn-treated *Atp13a2* mice	(14)
AAV rat	AAV human WT *ATP13A2* and alphaSyn overexpression	-Increased apomorphine rotation in alphaSyn rats	-Loss of TH-positive neurons in SNc and Str -Reduced DA and metabolites in Str	(13)

aSyn, alpha-synuclein; BMP, bis(monoacylglycerol)phosphate; BS, brain stem; CBL, cerebellum; CTX, cortex; DA, dopamine; GFAP, glial fibrillary acidic protein; HPC, hippocampus; Iba-1, ionized calcium-binding adaptor molecule 1; LAMP1/LAMP2, lysosome associated membrane protein-1 and -2; Mn, manganese; SNc, substantia nigra pars compacta; Str, striatum; TH, tyrosine hydroxylase; THL, thalamus.

Excess exposure to the heavy metals implicated in ATP13A2 function all negatively impact mitochondrial function (Tables 2, 3). Alaimo et al. (110) showed that Mn can cause dysregulation of fusion and fission, processes important in mitochondrial dynamics. An imbalance of these systems can result in ROS accumulation and cell death. Excess iron is also associated with mitochondrial dysfunction and increased ROS and is strongly linked to neurodegeneration (111). Zinc is shown to inhibit mitochondrial function causing increased ROS, energy impairments, and cytotoxicity (16, 17).

ATP13A2 transports polyamines out of the lysosome into the cytoplasm to maintain polyamine levels in cells (19, 112). Accumulation of polyamines is toxic as lysosomes can rupture when polyamine concentration is too high resulting in detrimental effects on the cell (64). *ATP13A2* mutations impair export of polyamines, resulting in lysosomal polyamine accumulation, reduced cytosolic polyamine levels and mitochondrial ROS cytotoxicity. Thus, ATP13A2 seems to be important in mediating polyamine levels which then further supports optimal mitochondrial function (64, 112).

### Lysosomal function

Similar to mitochondrial dysfunction, impaired protein degradation systems such as the autophagy lysosomal pathway underly multiple neurodegenerative diseases (113, 114). Autophagy lysosomal defects are prominent in PD, NCL, and Gaucher’s disease, among others. Early investigation into the effect of *ATP13A2* mutations on lysosomal function showed wildtype ATP13A2 localizes to the lysosome but that mutated ATP13A2 can localize to the endoplasmic reticulum (ER) causing ER stress and decreased lysosomal function (1, 12, 21, 50, 51, 115). Studies in *ATP13A2* patient-derived fibroblasts and in knockdown of *ATP13A2* in dopaminergic cell lines show multiple lysosomal anomalies including reduced degradation of lysosomal substrates, alterations in acidification, decreased clearance of autophagosomes, and impaired proteolytic processing of lysosomal enzymes (113). In mice, loss of *Atp13a2* function results in enhanced lipofuscinosis, accumulation of the substrates p62, cathepsin D, and ubiquitin (Table 4) (6, 104). ATP13A2 is also important for exosome secretion, where loss of function is associated with decreased exosomes, and overexpression promotes exosomal generation, release, and functioning (115). Collectively, impaired ATP13A2 function is linked to lysosome dysfunction, impaired exosome secretion, and autophagic flux (Table 4) (1, 6, 12, 21, 50, 51, 104, 113, 115).

### ATP13A2 and alpha-synuclein

In conjunction with mitochondrial and lysosomal defects, loss of *ATP13A2* function is shown to increase alpha-synuclein accumulation (52, 107, 108, 113, 116–118). Alpha-synuclein is a presynaptic protein involved in synaptic transmission, vesicular trafficking, and plasticity and it is the major component of Lewy bodies, the hallmark pathology in PD, Multiple System Atrophy, and Dementia with Lewy Bodies (119, 120). Studies show ATP13A2 is involved in the exosomal externalization of alpha-synuclein (Figure 1), indicating a potentially important role in PD and other synucleinopathies (16, 22). While the *in vitro* work establishing a relationship between loss of function of *ATP13A2* and alpha-synuclein is compelling, *in vivo* studies paint a more inconsistent picture (Table 4) (6, 13–15, 104, 113, 116). Differential effects are observed in *Atp13a2* null (13a2) mouse lines, as one study found abnormal alpha-synuclein accumulation in the brain while the other did not (6, 104). The mouse line with increased abnormal alpha-synuclein in the brain also exhibited increased triton-insoluble alpha-synuclein in the ventral midbrain in response to systemic manganese administration and enhanced sensorimotor deficits when combined with alpha-synuclein overexpression (Table 4) (14, 105). However, no acceleration of pathology was observed when a mutated form of alpha-synuclein (A53T) was overexpressed (104). In addition, viral co-overexpression of *Atp13a2* and alpha-synuclein did not protect against alpha-synuclein toxicity in the substantia nigra in rats (13). There are several methodological differences between the studies to note though including the timing (*Atp13a2* may need to precede alpha-synuclein overexpression) and level of overexpression of *Atp13a2*. In viral vector studies and in crossbreeding studies the promoter and type of alpha-synuclein being expressed (mutated or wildtype) are known to yield differential phenotypes and pathology (14). Clinically, the one postmortem case of KRS did not show Lewy body pathology (34). However, this is not unprecedented as other genetic forms of PD such as *LRRK2* have cases with Lewy body pathology and without (121–125). *ATP13A2* variants are common in *LRRK2* carriers and may modify disease onset and progression (8). More *in vivo* studies are needed to elucidate the relationship between ATP13A2 and alpha-synuclein.

ATP13A2’s role in polyamine transport, lysosomal function, and mitochondrial function suggests that when its function is impaired it leaves the basal ganglia particularly vulnerable to different types of insults be it heavy metal toxicity or alpha-synuclein toxicity. Understanding how these interactions develop and lead to basal ganglia dysfunction and neurodegeneration would inform multiple basal ganglia conditions and identify much needed novel targets for therapy.

## Author contributions

KC and SF conceptualized and wrote this work. All authors edited and approved the final version of the manuscript.
